# Mixing Test in a Case of Combined Acquired Hemophilia A and Coagulation Factor Deficiency: A Case Report

**DOI:** 10.7759/cureus.97307

**Published:** 2025-11-20

**Authors:** Osamu Imataki, Keigo Takahata, Yuko Mukai, Masayo Tamura, Akira Kitanaka

**Affiliations:** 1 Laboratory Medicine, Kawasaki Medical School, Okayama, JPN

**Keywords:** acquired hemophilia a, activated partial thromboplastin time (aptt), coagulation factor deficiency, coagulopathy, mixing test, prothrombin time (pt)

## Abstract

Acquired hemophilia A and B are rare autoimmune disorders where antibodies inhibit coagulation factors VIII and IX, leading to excessive bleeding. Acquired hemophilia displays an *inhibitor pattern* in mixing tests. Meanwhile, coagulation factor deficiency, often due to liver disease, results from reduced synthesis of coagulation factors and shows a *deficiency pattern* in mixing tests. However, overlapping features can complicate differentiation. A *percent correction *method in the mixing test improved accuracy in distinguishing between the *inhibitor pattern* and the *deficiency pattern*. Using this method, a complex case involving both acquired hemophilia A and liver-related coagulation factor deficiency was successfully diagnosed.

A 70-year-old man presented with subcutaneous, intracranial bleeding, and gastrointestinal hemorrhage. The laboratory data revealed severe anemia (hemoglobin 5.7 g/dL) without thrombocytopenia. Coagulation studies showed significantly prolonged activated partial thromboplastin time (APTT >100 seconds), mildly prolonged prothrombin time (PT 13.0 seconds), and a low fibrinogen (144 mg/dL). Given the disproportionately prolonged APTT, we performed a 1:1 *percent correction* of the mixing test, which revealed a *deficiency pattern* before treatment. We administered fresh frozen plasma (FFP); however, the coagulopathy was not improved. After the FFP treatment, the mixing test revealed a *deficiency pattern* in the early phase and an *inhibitor pattern* in the late phase. The coagulation factors were nonspecifically decreased. The inhibitor for coagulation factor VIII was identified. We diagnosed the patient with acquired hemophilia.

Diagnosing coagulopathy is especially challenging when a patient has both acquired hemophilia and coagulation factor deficiency, as mixing test patterns may overlap and lose distinct characteristics. The 1:1 *percent correction* method proved to be the most reliable approach for distinguishing between deficiency and inhibitor-related patterns. In this case, acquired hemophilia A was the primary cause of severe bleeding with underlying coagulation factor deficiency. Combining mixing tests with plasma supplementation helped clarify the dual pathogenesis.

## Introduction

Acquired hemophilia A and B are rare autoimmune disorders in which the body produces antibodies against clotting factors VIII and IX, respectively. This leads to excessive bleeding [1,2]. Coagulation factor deficiency, on the other hand, is characterized by an insufficient amount of one or more clotting factors necessary for proper blood clotting [3-5]. The *mixing test*, also known as the *cross-mixing test*, is a diagnostic test used to determine the cause of abnormal clotting times, particularly when prothrombin time (PT) or activated partial thromboplastin time (APTT) is prolonged [6]. It involves mixing patient plasma showing abnormal clotting with normal pooled plasma from a healthy donor to correct the clotting time to normal levels [7]. When plotting clotting time against patient plasma concentration, the shape of the resulting curve reveals the underlying cause of prolonged clotting. The *correction* curve is categorized into two patterns in a graph of clotting time as normal plasma is supplemented. The *deficiency pattern* is a *concave downward* curve, which means that normal plasma supplies the missing factors, improving the clotting time. The *inhibitor pattern* shapes a *convex upward* curve. This indicates that the patient's plasma contains substances that interfere with clotting, even when diluted [8]. The immediate mixing test captures the early response and measures clotting time right after mixing. In contrast, the incubated *mixing test* demonstrates a late response after incubating the mixture at 37 °C for one to two hours. This helps detect time-dependent inhibitors, such as inhibitors of coagulation factors.

Acquired hemophilia is a hemorrhagic disease that manifests as subcutaneous hemorrhage or hemarthrosis and shows an *inhibitor pattern* in the mixing test. Antiphospholipid syndrome, such as the detection of lupus anticoagulants, is another disease that shows an *inhibitor pattern* in the mixing test. Coagulation factor deficiency is seen in liver dysfunction, such as chronic hepatitis and liver cirrhosis. In this disease, the mixing test shows a *deficiency pattern*, and the coagulopathy index (PT or APTT) is retrieved by adding healthy plasma. The deficiency stems from impaired hepatic production of coagulation proteins, such as coagulation factors. A substantial proportion of patients with acquired hemophilia A have underlying diseases, such as malignancies, autoimmune diseases, post-infectious conditions, and certain drug reactions [7,9]. One of the underlying diseases associated with acquired hemophilia A is chronic liver disease [9]. Therefore, concomitant diseases of acquired hemophilia and coagulation factor deficiencies, such as chronic liver disease, are clinically confusing [7]. A rapid clinical decision is required to manage critical bleeding before the presence of an inhibitor is proven [7].

In cases involving the *inhibitor pattern* and *deficiency pattern* in the mixing test, the two patterns cannot be differentiated due to the loss of their respective typical forms: convex upward or concave downward, respectively. These two entities are often confused in the laboratory because they can both prolong the APTT, but behave differently in a mixing test. The central clinical problem is how to differentiate the coexistence of the two disorders, and we should foreshadow the solution, rapid diagnosis by repeated mixing tests plus factor supplementation. These two patterns should be distinguished visually; however, a recent report advocated for clear, distinct criteria to diagnose the two patterns [6]. The *percent correction* formula is an objective diagnostic tool that distinguishes between the two patterns [6]. The *mixing test* of patient plasma and normal pooled plasma at a 1:1 ratio measures whether adding normal pooled plasma immediately normalizes the clotting time. Failure of normalization after incubation suggests the presence of an inhibitor. In this literature, the authors provided a *percent correction* formula for the distinct and accurate evaluation of mixing test results.

We diagnosed a case of acquired hemophilia A accompanied by chronic liver dysfunction and coagulation factor deficiency using the *percent correction* method. In our patient, abnormal APTT was used to perform a mixing test, and we had to determine two possible patterns, which sometimes need to be interpreted with a numerical index. In this report, we demonstrate that the mixing test optimally discriminates between the two categories of coagulation factor dysfunction and provides learning points to help readers make a rapid diagnosis of coagulation diseases.

## Case presentation

We examined a 70-year-old male patient with coagulopathy, presenting with bleeding tendencies in the subcutaneous tissue and intracranial region. The patient was transferred to our hospital due to gastrointestinal bleeding. He had a medical history of schizophrenia for more than four decades, since the age of 27, which was managed with appropriate medication and did not interfere with his daily life. One year before this admission, the patient had been treated for a putaminal hemorrhage at the age of 69. At that time, his coagulation and fibrinolysis function was intact (PT 11.9 seconds, PT-INR 1.07, APTT 88.7%). The putaminal hemorrhage episode was resolved with conservative treatment.

At the onset of the bleeding tendency, the patient did not complain of any bleeding symptoms. A physical examination revealed pale skin, but his vital signs were stable: blood pressure 108/51 mmHg, pulse 90 beats per minute, body temperature 38.1 °C, respiration rate 20 breaths per minute, and SpO_2_ 100% (room air). The patient was conscious and showed no signs of motor or sensory paralysis. There were no abnormal findings in his chest or abdomen. Cutaneous purpura and mucosal bleeding tendencies were subclinical. The patient’s activity was limited to daily activities due to anemic symptoms. Laboratory data showed severe anemia with no thrombocytopenia. Table 1 shows the other routine clinical laboratory results used to screen for underlying diseases. Notably, the patient's coagulation function tests, including PT and APTT, were abnormal upon admission. The APTT was markedly prolonged rather than the PT. This is considered the cause of the patient's bleeding tendency. Additionally, impaired hepatic synthetic function was found; nevertheless, tests for hepatitis B surface antigen (HBsAg) and hepatitis C virus antibody (HCV-Ab) were negative. To screen for external pathway coagulation abnormalities, we performed a mixing test. An automated hemostasis analyzer, CN-6000 (Sysmex, Kobe, Japan), was used for the mixing test. And we used the reagents, Revohem APTT-SLA (Cat# BN125488; Sysmex) and Revohem 0.025M ClCa (Cat# AB200022; Sysmex). We incubated samples for 120 minutes to assess late APTT time. We indicated the two *mixing test* patterns and their interpretations in Table 2.

**Table 1:** Patient’s laboratory data at the disease onset. MCV, mean corpuscular volume; MCH, mean corpuscular hemoglobin; MCHC, mean corpuscular hemoglobin concentration; CRP, C-reactive protein; TP, total protein; Alb, albumin; BUN, blood urea nitrogen; CRE, creatinine; UA, uric acid; T-Bil, total bilirubin; AST, aspartate aminotransferase; ALT, alanine aminotransferase; ALP, alkaline phosphatase; LDH, lactate dehydrogenase; gGTP, gamma-glutamyl transpeptidase; Na, sodium; K, potassium; Cl, chloride; Ca, calcium; PT, prothrombin time; PT activity, prothrombin time activity; PT-INR, prothrombin time-international normalized ratio; APTT, activated partial thromboplastin time; FIB, fibrinogen; ATIII, antithrombin III

Count blood cells			Reference range
WBC	13,640	/µL	3,300-8,600
Band cells	1.0	%	2.0-10.0
Segmented cells	95.0	%	50.0-70.0
Eosinophil	0.0	%	1.0-5.0
Basophil	0.0	%	0.0-1.0
Lymphocyte	2.0	%	20.0-40.0
Monocyte	1.0	%	1.0-6.0
Metamyelocyte	1.0	%	0.0
Myelocyte	0.0	%	0.0
Promyelocyte	0.0	%	0.0
RBC	172 × 10^4^	/µL	435-555
Hemoglobin	5.7	g/dL	13.7-16.8
Hematocrit	16.5	%	40.7-50.1
MCV	95.9	fL	83.6-98.2
MCH	33.1	pg	27.5-33.2
MCHC	34.5	%	31.7-35.3
Platelet	18.7 × 10^4^	/µL	15.8-34.8
Biochemistry			
CRP	9.51	mg/dL	<0.14
TP	4.3	g/dL	6.6-8.1
Alb	1.1	g/dL	4.1-5.1
BUN	11.0	mg/dL	8-20
CRE	0.42	mg/dL	0.65-1.07
UA	2.5	mg/dL	3.7-7.8
T-Bil	0.5	mg/dL	0.4-1.5
AST	29	U/L	13-30
ALT	29	U/L	10-42
ALP	106	U/L	38-113
LDH	200	U/L	124-222
gGTP	9	U/L	13-64
Na	128	mmol/L	138-145
K	3.4	mmol/L	3.6-4.8
Cl	97	mmol/L	101-108
Ca	9.0	mg/dL	8.8-10.1
Coagulation/fibrinolysis			
PT	13.0	sec	9.3-12.5
PT activity	79.5	%	80.7-125.2
PT-INR	1.14		0.85-1.13
APTT	>100.0	sec	24.0-34.0
FIB	144	mg/dL	200-400
ATIII	49.1	%	80.0-130.0
D-Dimer	6.8	µg/mL	<1.0
Others			
vWF activity	296	%	60-170
vWF Ag	204	%	50-155
Lupus anticoagulant	1.2		0.0-1.2
b2GPIAb	≤1.2	U/mL	<3.5
APL panel			
Anti-CL IgG	3.8	U/mL	<20
Anti-CL IgM	1.1	U/mL	<20
Anti-GP1 IgG	<6.4	U/mL	<20
Anti-GP1 IgM	<1.1	U/mL	<20

**Table 2:** Diagnostic interpretation of the mixing test. A *percent correction* is a percent corrected value before and after the supplementation of the given normal plasma.

	Inhibitor present	Factor deficiency
Cause of coagulopathy	Coagulation factor inhibitor	Hemophilia A (factor VIII deficiency), vitamin K deficiency, and coagulation factor deficiency
Representative disease (incidence)	Acquired hemophilia A/B (1.5 cases per million a year) [9]	Liver cirrhosis (Coagulopathy is 5% in cirrhosis patients.)
Mixing test pattern	Inhibitor pattern	Deficiency pattern
Mixing test shape	Convex upward	Concave downward
Interpretation	No correction [8]	Correction occurs

We performed a 1:1 *percent correction* of the mixing test [5] with the patient data, which revealed that it met the criteria of the *deficiency pattern* of the mixing test before treatment:

Early phase: (170.5-42.9)/(170.5-26.9)=88.9% (>70%)

Late phase: (246.4-64.2)/(246.4-29.1)=83.8% (>70%)

The results met the criteria for a *deficiency pattern* diagnosis (1:1 percent correction >70%). These results suggest that the cause of this patient’s coagulopathy is coagulation factor inefficiency. According to the results of the mixing test, we evaluated the coagulation factors (Table 3) and anticoagulation factor antibodies (Table 4). Screening of the coagulation factors revealed diminished levels of all factors, with FVIII, IX, XI, XII, and XIII showing the most significant decrease (below 30%). The CT imaging did not reveal the findings of chronic hepatitis and massive hepatic organic lesions. However, the multiple parameters (albumin, fibrinogen, ATIII, coagulation factors) indicated an impaired hepatic synthetic function. FIB-4 index was 2.04 (≤1.3).

**Table 3:** Patient’s coagulation factors

Coagulation factors	activity
Factor II (F2)	52%
Factor V (F5)	61%
Factor VII (F7)	68%
Factor VIII (F8)	<1%
Factor IX (F9)	29%
Factor X (F10)	61%
Factor XI (F11)	<22%
Factor XII (F12)	<10%
Factor XIII (F13)	24%

**Table 4:** Patient’s anticoagulation factor inhibitors The detection limit is approximately 0.5 Bethesda Units per milliliter (BU/mL) [10].

Anti-coagulation factor antibody	(inhibitor)
Factor VIII inhibitor	(+)
Activity	92 BU/mL
Factor IX inhibitor	(-)
Activity	1 BU/mL

Based on this initial evaluation, we administered fresh frozen plasma (FFP) supplementation (FFP 2-4 U/day, for 8 days) as the initial treatment. However, after FFP therapy, the coagulopathy represented by prolonged APTT was not improved. After the FFP treatment, we re-evaluated the mixing test. The second evaluation of the *percent correction* of the mixing test [6] revealed a *deficiency pattern* (1:1 percent correction >70%) in the early phase and an *inhibitor pattern* (1:1 percent correction <58%) in the late phase.

Early phase: (186.1-43.1)/(186.1-27.1) = 89.9% (>70%)

Late phase: (171.7-93.5)/(171.7-29.0) = 54.8% (<58%)

**Figure 1:**
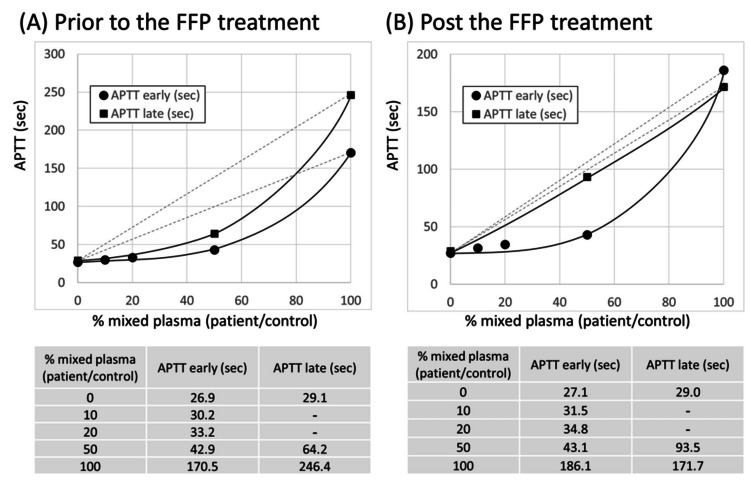
Mixing test results. A cross-mixing test was performed. (A) At the onset of acquired hemophilia A, a mixing test showed a convex downward form, *deficiency pattern*,* *both in the immediate and after incubation reaction. (B) After supplementation of coagulation factors by the treatment of fresh frozen plasma, a mixing test showed a convex upward form, *inhibitor pattern* only after incubation. *An automated hemostasis analyzer CN-6000 (Sysmex, Kobe, Japan) was used for the mixing test. And we used the reagents, Revohem APTT-SLA (Cat# BN125488) (Sysmex) and Revohem 0.025M ClCa (Cat# AB200022; Sysmex). The incubation time for late APTT assessment was 120 minutes. **1:1 percent correction ((a-c)/(a-d)) × 100, in which the APTT values (seconds) of a) neat test plasma, b) mixture of 4 parts test plasma to 1 part NPP, c) mixture of 1 part test plasma to 1 part NPP, d) NPP. The *deficiency pattern* diagnosis is made by a 1:1 *percent correction *>70% in the early phase. The *inhibitor pattern* diagnosis is made by a 1:1 *percent correction* <58% in the late phase. FFP, fresh frozen plasma; NPP, normal pooled plasma; APTT, activated partial thromboplastin time

**Figure 2:**
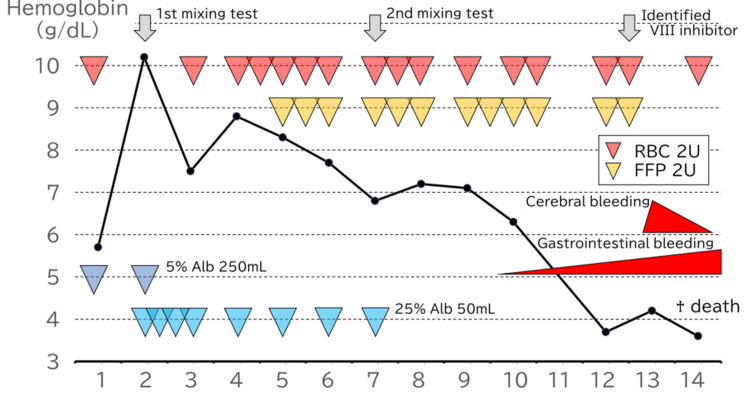
The clinical outline of the patient. We provided a clinical overview of the patient. After initiating treatment, we obtained the results of coagulation factor activity and the presence of an inhibitor and its titer. Throughout the treatment course, we sequentially suspected, differentiated, and diagnosed the patient's pathogenesis. The APTT remained prolonged and outside the normal range throughout the clinical course, even after FFP supplementation. Alb, albumin; FFP, fresh frozen plasma; APTT, activated partial thromboplastin time

Then, we screened the inhibitor for coagulation factors VIII and IX, identifying an inhibitor of factor VIII. We diagnosed the patient with acquired hemophilia A and an underlying coagulation factor deficiency. We did not follow the coagulation factor activity after the definite diagnosis. The patient was finally diagnosed with acquired hemophilia A and a deficiency in coagulation factors. Figure 1 shows the patient's mixing test. We indicated the clinical outline of the patient in Figure 2. The patient subsequently developed uncontrolled gastrointestinal bleeding and was complicated by cerebral bleeding. The patient passed away on the second day after the diagnosis of acquired hemophilia.

## Discussion

A differential diagnosis of coagulopathy using a mixing test is difficult if an individual has two coagulation disorders, such as acquired hemophilia and coagulation factor deficiency. The final diagnosis should be made based on clinical findings, including objective laboratory data. However, the initial assessment of coagulopathy using the mixing test is important for planning subsequent examinations and rapid critical interventions. In our case, we initially supplemented the patient with FFP based on the initial mixing test assessment. We specified the diagnostic test and identified the combined pathogenicity based on the results of the second mixing test after the FFP supplementation (Figure 2). The final diagnosis was made the day before the patient’s death when an inhibitor of a specific coagulation factor was detected. The two distinct patterns in the mixing test, *inhibitor* and *deficiency*, are represented in a combined and modified form. Even in such cases, the decision-making criteria to differentiate between the two patterns are essential. We used the *percent correction* method of the mixing test to distinguish these two patterns of coagulation disorders, as shown in the results section. Another scoring method is also useful for discriminating between the two coagulation mechanisms [11]. Matsuda et al. reported a scoring system that distinguishes inhibitors using a correction index for coagulation factor deficiency. We applied these five indices to interpret the mixing test in our patient (Table 5). Only the *1:1 percent correction* correctly reflects the patient’s pathogenesis.

**Table 5:** Five numerical indices to interpret the patient’s mixing test. Each index was calculated as follows: ICA = ((c-d)/a) × 100, mixing test normalized ratio = c/d, 1:1 percent correction ((a-c)/(a-d)) × 100, 4:1 percent correction = ((a-b)/(a-d)) × 100, Mix–NPP = c-d, in which the APTT values (seconds) of a) neat test plasma, b) mixture of four parts test plasma to one part NPP, c) mixture of one part test plasma to one part NPP, d) NPP. The cutoff values used were determined as described in previous studies. The gray column indicates the matched results with clinical diagnosis. Boldface is used to mark concordant results. APTT, activated partial thromboplastin time; ICA, index of circulation anticoagulant; Mix, 1:1 mixture; NPP, normal pooled plasma

	Initial evaluation		After FFP therapy		Cutoff value for factor deficiency
	Early	Late	Early	Late	
ICA (Rosner Index)	9.38	14.2	8.60	37.6	<12.4%
Mixing test normalization ratio	1.59	2.21	1.59	3.22	<1.12
1:1 percent correction	88.9	83.8	89.9	54.8	≥72%
4:1 percent correction	52.6	46.7	61.1	25.0	≥50%
Mix-TTP	16.0	35.1	16.0	64.5	<8 seconds

The circulation anticoagulant index (ICA) [12], the normalized ratio [13], and the Mix-NPP [14] are specifically produced indices that normalize the results of the anti-lupus coagulant mixing test. Meanwhile, the simple *percent correction* is sensitive enough to detect coagulation factor deficiency. ICA accurately indicates the presence of circulating anticoagulants in incubated samples before and after FFP therapy. As in our case, if a patient is diagnosed with a *deficiency* pattern using the mixing test and the coagulopathy does not recover after proper supplementation with coagulation factors such as FFP, the possibility of an inhibitor-related coagulopathy should be considered. After FFP supplementation, the mixing test should be examined again [15]. This is reasonable because the prolonged clotting time of defibrinating normal plasma was *corrected* by the mixing study in more than 90% of cases. However, the prolonged clotting time of plasma with inhibitors was *non-corrected* in half of the cases and *partially corrected* in the other half by the mixing study [15]. In other words, *correction* generally indicates coagulation factor deficiency, whereas *non-correction* suggests the presence of an inhibitor. In any case, the *1:1 percent correction* method is a simple, predictive way to differentiate the influence of coagulation factor deficiency [5].

Although no case reports combining both conditions were identified, there have been instances in which patients with acquired hemophilia A presented with other coagulation disorders. These cases are extremely rare and require specialized medical management. Combinatory conditions of autoimmune-mediated coagulopathy induced by inhibitors (i.e., acquired hemophilia) and coagulation factor deficiencies (e.g., liver cirrhosis, drug-induced, and malnutrition) have been reported in a few case reports in the literature [16-19]. The most frequent combination is antiphospholipid syndrome and coagulation factor deficiency [16-19]. In all cases of this combination of illnesses, circulating antibodies to coagulation factors, in addition to coagulation deficiency, are the causative pathogenesis of coagulopathy. In that case, normal plasma supplementation can partially or completely correct the *deficiency pattern* [20]. A mixing test can be used to correct coagulation factor deficiencies by mixing samples with an equal volume of normal plasma. After determining the effect of the deficiency, we can accurately evaluate the *inhibitor pattern*.

The patient's history of putaminal hemorrhage suggested chronic coagulation factor deficiency, which was not reflected by abnormal PT or APTT. In the current episode of bleeding tendency, the patient presented with acquired hemophilia A, which, when combined with the coagulation factor deficiency, induced symptoms of spontaneous internal bleeding. We detected a nonspecific reduction in coagulation factors VIII-IX and XI-XIII. Other coagulation factors, such as fibrinogen, also decreased. Von Willebrand factor was intact. Anti-phospholipid antibodies, including anti-β2GPI, anti-cardiolipin, and anti-GP1, were all negative. Thus, the onset of acquired hemophilia A was the definitive determinant of major bleeding, specifically intracranial and gastrointestinal bleeding, in this patient's clinical course.

## Conclusions

In summary, our case notified coexistence of acquired hemophilia and coagulation factor deficiency. It is difficult to differentiate the etiology of a concomitant disease involving two coagulopathies: coagulation factor deficiency and circulating coagulation inhibitors. As shown in our case, diagnosing coagulopathy is especially challenging when a patient has both acquired hemophilia and coagulation factor deficiency, as mixing test patterns may overlap and lose distinct characteristics. The repeated mixing test with the *percent correction *method is optimal to clarify the dual pathogenesis.

## References

[1] Hay CR, Brown S, Collins PW, Keeling DM, Liesner R (2006). The diagnosis and management of factor VIII and IX inhibitors: a guideline from the United Kingdom Haemophilia Centre Doctors Organisation. Br J Haematol.

[2] Rodeghiero F, Tosetto A, Castaman G (2007). How to estimate bleeding risk in mild bleeding disorders. J Thromb Haemost.

[3] Tripodi A (2015). Hemostasis abnormalities in cirrhosis. Curr Opin Hematol.

[4] Casini A, Gebhart J (2024). How to investigate mild to moderate bleeding disorders and bleeding disorder of unknown cause. Int J Lab Hematol.

[5] Mehic D, Gebhart J, Pabinger I (2024). Bleeding disorder of unknown cause: a diagnosis of exclusion. Hamostaseologie.

[6] Chang SH, Tillema V, Scherr D (2002). A "percent correction" formula for evaluation of mixing studies. Am J Clin Pathol.

[7] Tiede A, Collins P, Knoebl P (2020). International recommendations on the diagnosis and treatment of acquired hemophilia A. Haematologica.

[8] W Collins P, Chalmers E, Hart D (2013). Diagnosis and management of acquired coagulation inhibitors: a guideline from UKHCDO. Br J Haematol.

[9] Lehoczki A, Fekete M, Mikala G, Bodó I (2025). Acquired hemophilia A as a disease of the elderly: a comprehensive review of epidemiology, pathogenesis, and novel therapy. Geroscience.

[10] (1975). Letter: A more uniform measurement of factor VIII inhibitors. Thromb Diath Haemorrh.

[11] Matsuda M, Hoshiyama Y, Ogawa K, Emmi M, Terai S, Moriyama M (2023). Performance characteristics of 5 numerical indexes in mixing test interpretation under coexistence of lupus anticoagulant and coagulation factor deficiency. Res Pract Thromb Haemost.

[12] Kumano O, Moore GW (2018). Lupus anticoagulant mixing tests for multiple reagents are more sensitive if interpreted with a mixing test-specific cut-off than index of circulating anticoagulant. Res Pract Thromb Haemost.

[13] Kershaw G, Orellana D (2013). Mixing tests: diagnostic aides in the investigation of prolonged prothrombin times and activated partial thromboplastin times. Semin Thromb Hemost.

[14] Cabo J, Morimont L, Baudar J (2023). Variability among commercial batches of normal pooled plasma in lupus anticoagulant testing. Int J Lab Hematol.

[15] Favaloro EJ (2020). Coagulation mixing studies: utility, algorithmic strategies and limitations for lupus anticoagulant testing or follow up of abnormal coagulation tests. Am J Hematol.

[16] Jin Y, Cheng Y, Mi J, Xu J (2023). A rare case of schizophrenia coexistence with antiphospholipid syndrome, β-thalassemia, and monoclonal gammopathy of undetermined significance. Front Psychiatry.

[17] Moore GW (2023). Lupus anticoagulant testing: dilute prothrombin time (dPT). Methods Mol Biol.

[18] Rodriguez V, Reed AM, Kuntz NL, Anderson PM, Smithson WA, Nichols WL (2005). Antiphospholipid syndrome with catastrophic bleeding and recurrent ischemic strokes as initial presentation of systemic lupus erythematosus. J Pediatr Hematol Oncol.

[19] Ayoub O, Aljurf M, Al Nounou R, Chaudhri NA (1999). Systemic lupus erythematosus presenting with haemorrhagic manifestation. Clin Lab Haematol.

[20] Pennings MT, De Groot PG, Meijers JC, Huisman A, Derksen RH, Urbanus RT (2014). Optimisation of lupus anticoagulant tests: should test samples always be mixed with normal plasma?. Thromb Haemost.

